# Effects of Salt Stress on Physiological and Agronomic Traits of Rice Genotypes with Contrasting Salt Tolerance

**DOI:** 10.3390/plants13081157

**Published:** 2024-04-22

**Authors:** Yunming Xu, Weicheng Bu, Yuchao Xu, Han Fei, Yiming Zhu, Irshad Ahmad, Nimir Eltyb Ahmed Nimir, Guisheng Zhou, Guanglong Zhu

**Affiliations:** 1 Joint International Research Laboratory of Agriculture and Agri-Product Safety, The Ministry of Education of China, Yangzhou University, Yangzhou 225009, China; mz120211307@stu.yzu.edu.cn (Y.X.); mz120231410@stu.yzu.edu.cn (H.F.); 2 Jiangsu Provincial Key Lab of Crop Genetics and Physiology, Yangzhou University, Yangzhou 225009, China; mx120220721@stu.yzu.edu.cn (Y.Z.); gszhou@yzu.edu.cn (G.Z.); 3 Jiangsu Co-Innovation Center for Modern Production Technology of Grain Crops, Yangzhou University, Yangzhou 225009, China; mz120231366@stu.yzu.edu.cn (W.B.); irshadgadoon737@yahoo.com (I.A.); 4 Jiangsu Yancheng Port Salty-Soil Agriculture Circular Agricultural Co., Ltd., Yancheng 224000, China; 17626672423@163.com; 5 Faculty of Agriculture, University of Khartoum, Khartoum 11115, Sudan; nimir1000@gmail.com

**Keywords:** growth traits, grain yield, physiological mechanism, rice, salt stress

## Abstract

Salinity is one of the major constraints to crop production. Rice is a main staple food and is highly sensitive to salinity. This study aimed to elucidate the effects of salt stress on physiological and agronomic traits of rice genotypes with contrasting salt tolerance. Six contrasting rice genotypes (DJWJ, JFX, NSIC, HKN, XD2H and HHZ), including three salt-tolerant and three salt-sensitive rice genotypes, were grown under two different salt concentrations (0 and 100 mmol L^−1^ NaCl solution). The results showed that growth, physiological and yield-related traits of both salt-sensitive and salt-tolerant rice were significantly affected by salt stress. In general, plant height, tiller number, dry weight and relative growth rate showed 15.7%, 11.2%, 25.2% and 24.6% more reduction in salt-sensitive rice than in salt-tolerant rice, respectively. On the contrary, antioxidant enzyme activity (superoxide dismutase, peroxidase, catalase), osmotic adjustment substances (proline, soluble protein, malondialdehyde (MDA)) and Na^+^ content were significantly increased under salt stress, and the increase was far higher in salt-tolerant rice except for MDA. Furthermore, grain yield and yield components significantly decreased under salt stress. Overall, the salt-sensitive rice genotypes showed a 15.3% greater reduction in grain yield, 5.1% reduction in spikelets per panicle, 7.4% reduction in grain-filling percentage and 6.1% reduction in grain weight compared to salt-tolerant genotypes under salt stress. However, a modest gap showed a decline in panicles (22.2% vs. 22.8%) and total spikelets (45.4% vs. 42.1%) between salt-sensitive and salt-tolerant rice under salinity conditions. This study revealed that the yield advantage of salt-tolerant rice was partially caused by more biomass accumulation, growth rate, strong antioxidant capacity and osmotic adjustment ability under salt stress, which contributed to more spikelets per panicle, high grain-filling percentage and grain weight. The results of this study could be helpful in understanding the physiological mechanism of contrasting rice genotypes’ responses to salt stress and to the breeding of salt-tolerant rice.

## 1. Introduction

The development and utilization of saline soils has been paid more and more attention because cultivated areas are shrinking more quickly than ever due to environmental degradation and urbanization [1]. Globally, approximately 1 billion ha of land is now affected by salinity [2]. In China, about 100 million ha of land is salinized, over 50% of which can be used to develop crop production [3]. Saline soil is an important reserve land resource with great potential utilization value, so the improvement and utilization of saline lands for crop production has become a national strategy to ensure food security in China [4].

Rice (*Oryza sativa* L.) is one of the main staple foods for the human diet [5]. In China, the rice production area was 29 million hm^2^ in 2023 [6]. Of the cereal crops, rice is the most sensitive to salinity [7,8]. Globally, about one-third of rice production areas are affected by salinity [9]. Plant growth and crop production are greatly restricted under salinity conditions [10]; the variable responses of rice genotypes to salinity stress are dependent on their growth stages [11]). Several studies confirmed that rice is tolerant to salinity during the germination and vegetative stages but sensitive at the early seedling stage and reproductive stage [12]. Under salinity stress, plant growth, tiller number, biomass accumulation, panicle number, spikelets per panicle, grain-filling percentage and grain yield all significantly declined [13]. These decreases are attributed to ion toxicity, nutrient deficiency and oxidative stress caused by salinity conditions [14].

During the germination stage, seed water uptake is suppressed largely under salt stress, which leads to a low germination rate and poor seedling establishment [14]. When rice encounters salt stress at the seedling stage, the seedling growth is significantly limited and results in a low leaf area index (LAI) [15]. It was confirmed that the damage is even worse when rice suffers salt stress during the early tillering stage; both tiller formation and plant height are severely decreased, and it generates fewer productive panicles at maturity [16]. At the panicle initiation stage, salt stress gives rise to the maldevelopment of the young panicles, spikelets per panicle decline, and the date of heading and flowering is delayed or prolonged. However, at the heading stage, rice blooming and fertilization are affected by salt stress, which causes a large amount of unfilled grain and a low grain-filling percentage [11].

Salt-sensitive and salt-tolerant rice genotypes have very different responses to salt stress. In general, a high level of salinity in the soil causes an imbalance in osmotic potential, ionic equilibrium and nutrient uptake [17,18,19]. Ionic toxicity is caused by salt stress, resulting in a large accumulation of intracellular Na^+^, disrupting the original ionic balance, causing a nutrient deficiency, and stunting plant growth and development [20]. More sodium accumulates in salt-sensitive rice, which impairs a wide range of cellular metabolisms, including photosynthesis, protein synthesis and lipid metabolism [21,22]. Salt-tolerant rice has higher Na^+^ exclusion ability and greater sequestration capacity than sensitive genotypes, which can exclude salts from entering active leaves [11]. A decrease in chlorophyll content becomes a first indication of responses in plants subjected to salinity stress [23]. Salt-tolerant rice can retain a higher content of chlorophyll than sensitive genotypes, particularly in the upper leaves, which can maintain higher efficiency photosynthesis to produce more biomass and promote seedling growth. It was reported that leaves of salt-sensitive rice shrink and wither more seriously under salt stress, which brings about a great yield loss. However, compared with salt-sensitive rice, salt-tolerant rice can maintain better growth and development and a lower decrease in yield under salinity conditions [24].

Previous research mainly concentrated on the screening of salt-tolerant rice genotypes, investigating the physiological mechanisms of rice under salinity conditions, and how to improve salt tolerance [12,25,26,27]. However, there exists a large difference between salt-sensitive and salt-tolerant rice genotypes’ responses to salt stress. Therefore, a controlled study was conducted with six contrasting rice genotypes under salt stress. The purpose of this study was to elucidate the effects of salt stress on physiological and agronomic traits of contrasting rice genotypes with contrasting responses to salt stress.

## 2. Results

### 2.1. Growth Characteristics of Different Rice Genotypes under Salt Stress

**Figure 1:**
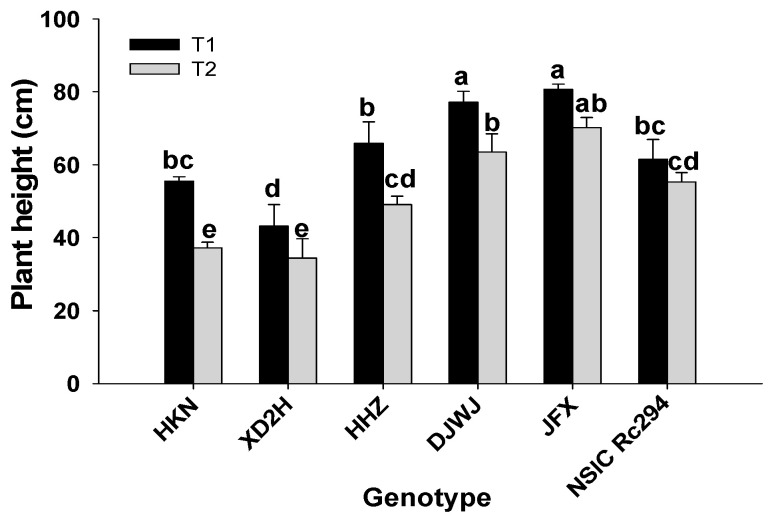
Effect of salt stress on plant height of different rice genotypes. T1: control, T2: salt treatment; Different lowercase letters indicate significant level of difference between genotypes under different treatments at *p* < 0.05. The plant height was measured with 10 plants in each replicate.

Salt stress had a significant effect on plant height of rice genotypes (*p* < 0.05, Table S1, Figure 1). The plant height was prominently decreased under salt stress, and the decrease was more severe in salt-sensitive rice than in salt-tolerant rice (Figure S1). Overall, salt-sensitive rice showed a 15.7% greater decrease in plant height than salt-tolerant rice under salt treatment. Among these, the salt-sensitive rice genotypes HKN and XD2H decreased by 31.8% and 19.1% more in plant height than salt-tolerant rice with genotype NSIC Rc294. On average, the plant height of salt-sensitive rice genotypes decreased by 25.3%; the highest decrease was 33.1% produced by HKN, followed by HHZ at 22.4%, and the lowest was 20.4% for XD2H. On the contrary, the plant height of salt-tolerant rice decreased by only 10.6% on average under salt stress; the smallest decrease was shown by NSIC Rc294 at 1.3%, followed by JFX and DJWJ at 12.9% and 17.8%, respectively (Figure 1).

**Figure 2:**
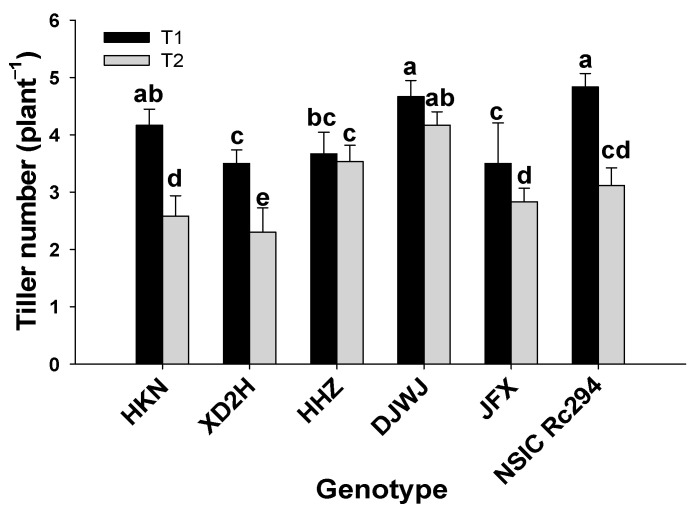
Effect of salinity stress on tiller number of different rice genotypes. T1: control, T2: salt treatment; Different lowercase letters indicate significant level of difference between genotypes under different treatments at *p* < 0.05. The tiller number was measured with 10 plants in each replicate.

The tiller number was significantly affected by salt stress (*p* < 0.05, Table S1, Figure 2), and the tiller number of all rice genotypes was obviously decreased to some extent under salt stress (Figure S2). Overall, salt-tolerant rice produced 11.2% higher tiller number than salt-sensitive rice under salt stress. In general, the tiller number of salt-sensitive genotypes decreased by 32.6% under salinity stress; the highest decrease was produced by HKN at 44.0%, followed by XD2H at 42.9% and HHZ with the lowest decrease of 11.1%. Similarly, the tiller number of salt-tolerant rice decreased by 21.4% under salt stress; both DJWJ and JFX showed a moderate decrease in tiller number under salinity stress, at 10.7% and 19.0%, respectively. However, as a salt-tolerant genotype, the tiller number of NSIC Rc294 showed the highest decrease at 34.4% (Figure 2).

Fresh weight was significantly affected by salt stress, genotype and the interaction between salt stress and genotype (*p* < 0.01 or 0.001, Table 1). In general, salt stress significantly decreased the fresh weight of all rice genotypes (Table S2). Compared with T1, the fresh weight of salt-tolerant rice decreased by 49.5% on average under salt stress, among which the decrease was 44.4% for DJWJ, followed by 47.2% and 56.9% for JFX and NSIC, respectively. However, the fresh weight of salt-sensitive rice was decreased by 41.4% on average, which was an 8.1% lower decrease than in the salt-tolerant rice genotypes. The highest decrease was shown by HKN at 57.6%, followed by HHZ at 44.0%, and the lowest was XD2H at 22.6% (Table 1).

Both dry weight and RGR were significantly affected by salinity stress and genotype (*p* < 0.05) but not by their interaction (*p* > 0.05, Table 1). The dry weight of all rice genotypes was prominently decreased under salt stress, and the decline was more severely shown by salt-sensitive genotypes (Table S2). Overall, in salt-sensitive rice, the dry weight decreased by 25.2% more than in salt-tolerant genotypes under salt treatment. Compared with the control, the dry weight of salt-tolerant rice decreased by 25.6% on average under salt stress, and the decrease was 9.9%, 32.4% and 34.5% for DJWJ, NSIC and JFX, respectively. In addition, the dry weight of salt-sensitive genotypes decreased by 50.8% on average; the highest decrease was produced by XD2H at 63.6%, followed by HKN at 51.1% and HHZ at 37.8% (Table 1).

**Figure 3:**
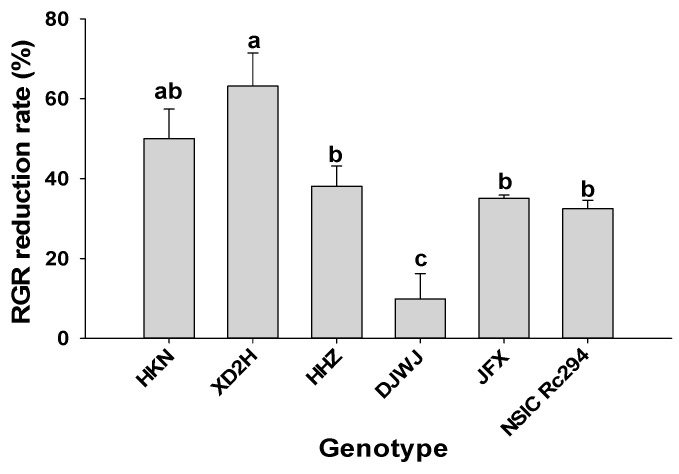
Effect of salt stress on relative growth rate (RGR) of different rice genotypes. Different lowercase letters indicate significant level of difference at *p* < 0.05. The RGR was measured with 10 plants in each replicate.

Similarly, RGR was significantly decreased under salt stress (*p* < 0.05, Table S2). On average, the RGR of salt-sensitive rice decreased by 50.4%, but salt-tolerant rice decreased by only 25.8% (Table 1, Figure S3). In salt-sensitive rice genotypes, the highest decrease was shown by XD2H at 63.2%, followed by HKN at 50.0% and HHZ at 38.1%. However, in salt-tolerant rice genotypes, the RGR of DJWJ only declined by 9.8%, and NSIC and JFX generated a comparative decrease in RGR, at 32.4% and 35.1%, respectively (Figure 3).

### 2.2. Physiological Characteristics of Different Rice Genotypes under Salt Stress

#### 2.2.1. Membrane Lipid Peroxidation

**Figure 4:**
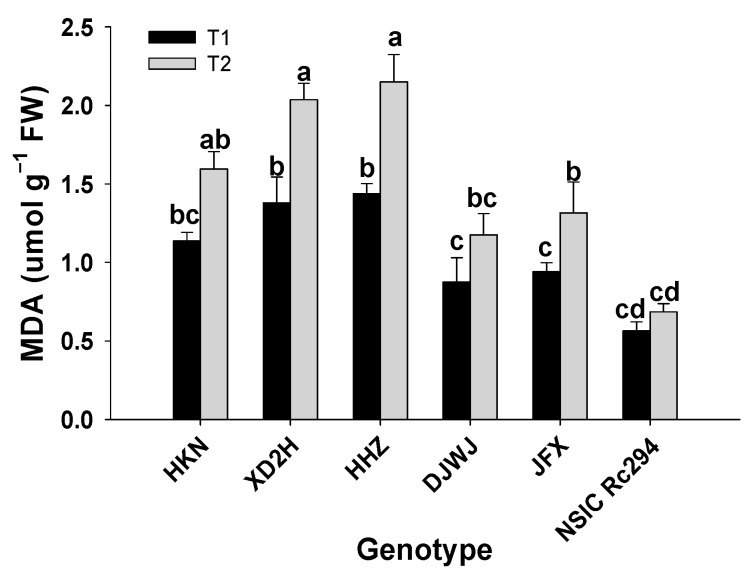
Effect of salt stress on MDA content of different rice genotypes. T1: control, T2: salt treatment; Different lowercase letters indicate significant level of difference between genotypes under different treatments (*p* < 0.05). The MDA was measured with frozen leaves by 5 repetitions in each replicate.

Salt stress had a significant effect on the MDA of rice genotypes (*p* < 0.05, Table S3, Figure S4), and the MDA content of each genotype was significantly increased under salt stress (*p* < 0.05, Figure 4). However, the variation of MDA was opposite to the above-mentioned physiological parameters, showing it was extremely higher in salt-sensitive rice than in salt-tolerant rice. On average, the MDA content of salt-sensitive genotypes increased by 45.7%; the highest increase was shown by HHZ at 49.4%, followed by XD2H at 47.7% and HKN at 40.1%. Furthermore, the MDA content of salt-tolerant genotypes increased by 31.7% under salt stress; JFX had the maximum increase at 39.5%, followed by DJWJ and NSIC at 34.2% and 21.3%, respectively. Overall, salt-sensitive genotypes produced 14.0% more MDA than salt-tolerant genotypes under salt treatment (Figure 4).

#### 2.2.2. Antioxidant Enzyme Activity

**Figure 5:**
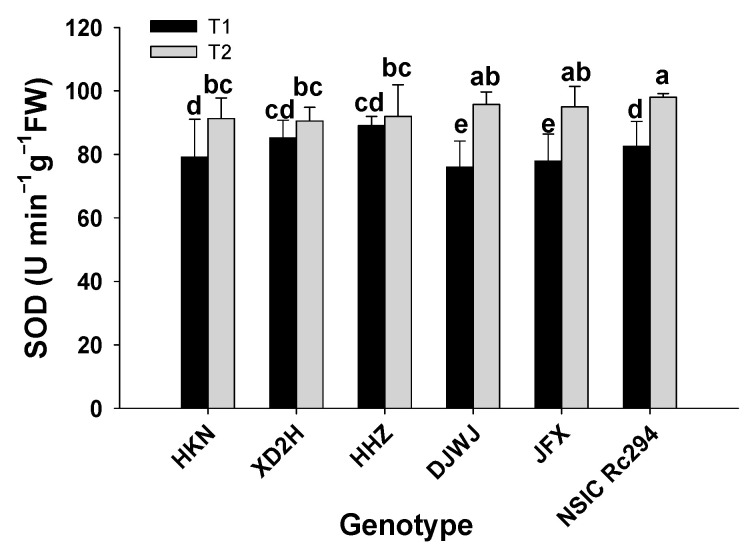
Effect of salt stress on SOD activity of different rice genotypes. T1: control, T2: salt treatment; Different lowercase letters indicate significant differences between genotypes under different treatments (*p* < 0.05). The SOD was measured with frozen leaves by 5 repetitions in each replicate.

The superoxide dismutase (SOD) activity was significantly affected by salt treatment, genotype, and their interaction (*p* < 0.05, Table S4, Figure S5). The SOD activities of both types of rice genotypes were significantly improved under salt stress (*p* < 0.05, Figure 5). The increase was more remarkable in salt-tolerant rice than in salt-sensitive rice. On average, the SOD activity of salt-tolerant rice increased by 22.2% under salt stress; the genotype DJWJ had the highest increase at 25.9%, followed by JFX and NSIC at 21.9% and 18.8%, respectively. However, the SOD activity of salt-sensitive rice increased by 8.3%; the genotypes HKN, XD2H and HHZ increased by 15.3%, 6.2% and 3.2%, respectively. Overall, salt-tolerant rice had 13.9% higher SOD activity than salt-sensitive rice under salt treatment (Figure 5).

**Figure 6:**
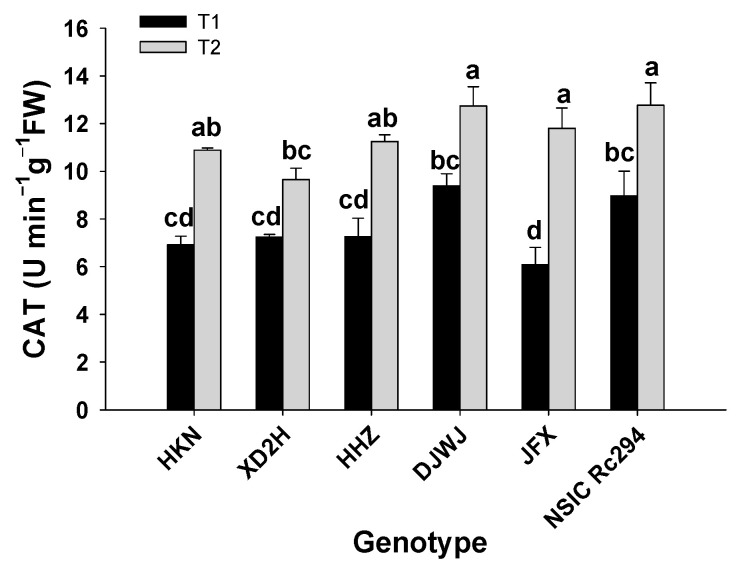
Effect of salt stress on CAT activity of different rice genotypes. T1: control, T2: salt treatment; Different lowercase letters indicate significant level of difference between genotypes under different treatments (*p* < 0.05). The CAT was measured with frozen leaves by 5 repetitions in each replicate.

Salt stress had a significant effect on the catalase (CAT) activity of rice genotypes (*p* < 0.05, Table S4, Figure S6). The CAT activity of each rice genotype increased prominently under salt stress (Figure 6). Overall, the increase was 10.3% higher in salt-tolerant rice than in salt-sensitive rice. Overall, compared with CK, the CAT activity of salt-tolerant rice increased by 58.8% under salt stress; JFX showed the highest increase at 98.6%, followed by NSIC and DJWJ at 42.2% and 35.7%, respectively. In striking contrast, the CAT activity of salt-sensitive rice increased by 48.5%; the highest increase was shown by HKN at 57.2%, followed by HHZ at 54.9%, and the lowest increase was 33.3%, produced by XD2H (*p* < 0.05, Figure 6).

**Figure 7:**
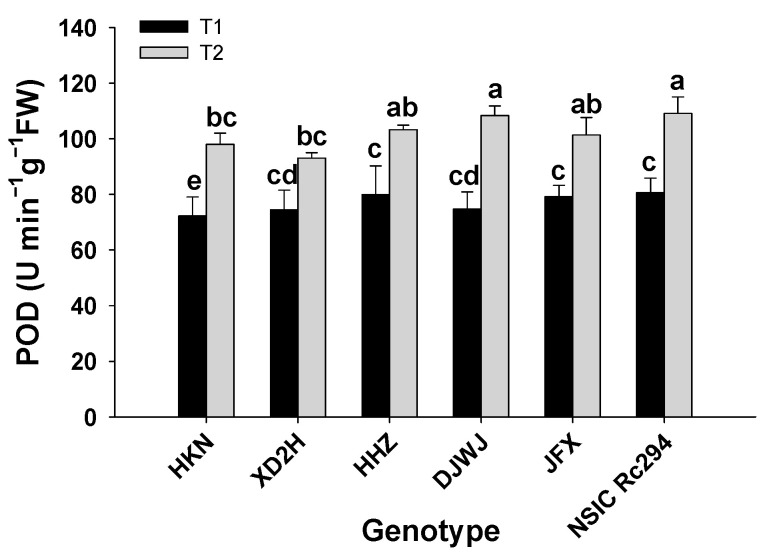
Effect of salt stress on POD activity of different rice genotypes. T1: control, T2: salt treatment; Different lowercase letters indicate significant level of difference between genotypes under different treatments (*p* < 0.05). The POD was measured with frozen leaves by 5 repetitions in each replicate.

The peroxidase (POD) activity of rice genotypes was significantly increased under salt stress (*p* < 0.05, Table S4, Figure S7). There was a significant difference in POD activity between salt-tolerant and salt-sensitive rice genotypes under salinity (Figure 7). On average, the POD activity of salt-tolerant rice increased by 36.0% after salt stress; the maximum increase was produced by DJWJ at 44.9%, followed by NSIC and JFX at 35.2% and 27.9%, respectively. However, the POD activity of salt-sensitive rice increased by 29.9%; HKN showed the highest increase at 35.6%, followed by HHZ at 29.2%, and XD2H at 25.0%. Overall, salt-sensitive rice genotypes showed 15.0% lower POD activity than salt-tolerant rice genotypes under salt treatment (Figure 7).

#### 2.2.3. Osmoregulatory Substances

**Figure 8:**
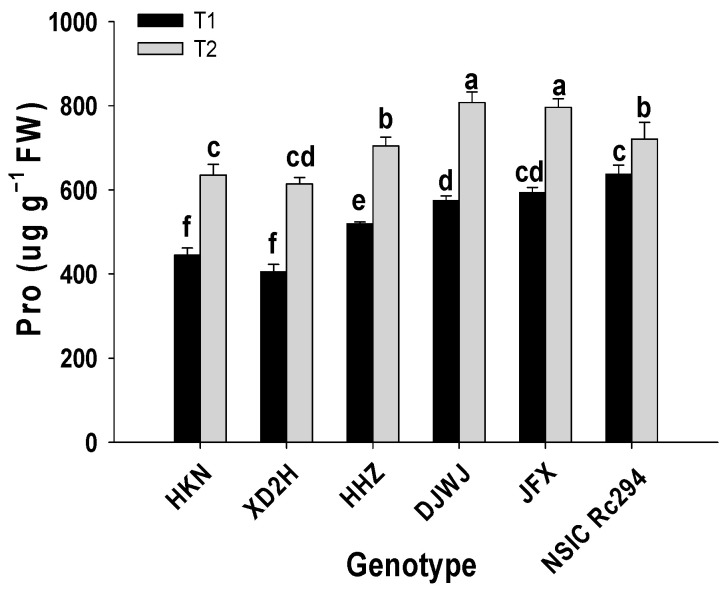
Effect of salt stress on Pro content of different rice genotypes. T1: control, T2: salt treatment; different lowercase letters indicate significant level of difference between genotypes under different treatments (*p* < 0.05). Pro content was measured with frozen leaves by 5 repetitions in each replicate.

Salt stress had a significant effect on the proline (Pro) content of rice genotypes (*p* < 0.05, Table S3, Figure 8), and the content of Pro was significantly increased under salt stress (*p* < 0.05, Figure 8). It was shown that the highest content of Pro was produced in salt-tolerant rice rather than in salt-sensitive rice. On average, the content of Pro in salt-sensitive genotypes increased by 43.4%; the highest increase was shown by XD2H at 51.7%, followed by HKN at 42.9% and HHZ at 35.5%. Furthermore, the content of Pro in salt-tolerant genotypes increased by 29.3% under salt stress; DJWJ performed the maximum increase at 40.6%, followed by JFX and NSIC at 34.2% and 13.2%, respectively (Figure 8).

**Figure 9:**
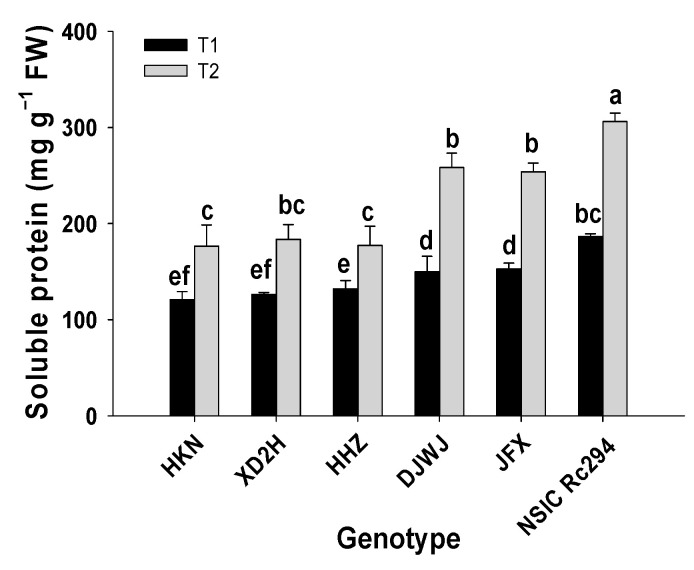
Effect of salt stress on soluble protein of different rice genotypes. T1: control, T2: salt treatment; Different lowercase letters indicate significant level of difference between genotypes under different treatments (*p* < 0.05). The soluble protein was measured with frozen leaves by 5 repetitions in each replicate.

The effect of salt stress on the soluble protein of rice genotypes was significant (*p* < 0.05, Table S3, Figure S8); the content of soluble protein greatly increased (*p* < 0.05, Figure 9). Consistently, the increase in soluble protein in salt-tolerant rice genotypes was greater than that in salt-sensitive genotypes under salinity. Viewed collectively, the soluble protein of salt-tolerant rice increased by 66.6% under salt stress; the maximum increase reached 72.5%, shown by DJWJ, followed by 66.1% and 64.1% increases for JFX and NSIC, respectively. In striking contrast, the soluble protein of salt-sensitive genotypes increased by 41.7%; HKN showed the highest increase at 46.1%, followed by XD2H at 45.2%, and the least increase was in HHZ at 33.9%. Overall, salt-tolerant genotypes generated a 24.9% higher increase in soluble protein than salt-sensitive genotypes under salt treatment (Figure 9).

#### 2.2.4. Na^+^, K^+^ and Na^+^/K^+^

The content of Na^+^ was significantly affected by salt treatment, genotype and their interaction (*p* < 0.05, Table 2). The content of Na^+^ in rice genotypes was significantly increased under salt stress. Compared with the control, the Na^+^ content of salt-tolerant genotypes increased by 411.9% on average; DJWJ showed the highest increase of 468.6%, followed by NSIC and JFX at 398.3% and 368.7%, respectively. The average Na^+^ content of salt-sensitive genotypes increased by 443.7%; XD2H increased by 470.8%, followed by HKN at 462.1% and HHZ with the lowest increase of 398.2%. Overall, the Na^+^ content of salt-sensitive genotypes increased by 32.8% more than in salt-tolerant genotypes under salt treatment (Table 2).

The content of K^+^ was significantly affected by salt treatment, genotype and their interaction (*p* < 0.05, Table 2). The content of K^+^ in rice genotypes was significantly decreased under salt stress. Compared with the control, the average K^+^ content of salt-tolerant genotypes decreased by 23.5% under salt stress, with DJWJ decreased by 16.9%, followed by NSIC and JFX at 22.5% and 31.2%, respectively. The average K^+^ content of salt-sensitive genotypes decreased by 39.3%; the largest decrease was in HHZ at 47.7%, followed by XD2H at 40.4%, and HKN with the lowest decrease of 29.7%. Overall, the K^+^ content of salt-sensitive genotypes decreased by 15.8% more than in salt-tolerant genotypes under salt treatment (Table 2).

The Na^+^/K^+^ content was significantly affected by salt treatment and genotype (*p* < 0.05) but not by their interaction (*p* > 0.05, Table 2). Compared with the control, the average Na^+^/K^+^ content of salt-tolerant genotypes increased by 570.6%; the highest increase was produced by DJW at 587.5%, followed by JFX and NSIC at 574.4% and 550.0%, respectively. The average Na^+^/K^+^ content of salt-sensitive genotypes increased by 805.8% on average; the maximum increase was shown by XD2H at 858.8%, followed by HHZ at 849.1% and HKN at 694.3%. Overall, the Na^+^/K^+^ content of salt-sensitive genotypes increased by 230.1% more than in salt-tolerant genotypes under salt treatment (Table 2).

### 2.3. Yield and Yield Components of Different Rice Genotypes under Salt Stress

Grain yield, spikelets per panicle, total spikelets and grain-filling percentage were significantly affected by salt stress, genotype and the interaction between salt stress and genotype (*p* < 0.05 or 0.001, Tables S5 and S6 and Table 3). However, panicles and 1000-grain weight were only affected by salt stress and genotype (*p* < 0.01 or 0.001), not by their interaction (*p* > 0.05, Tables S5 and S6 and Table 3).

**Figure 10:**
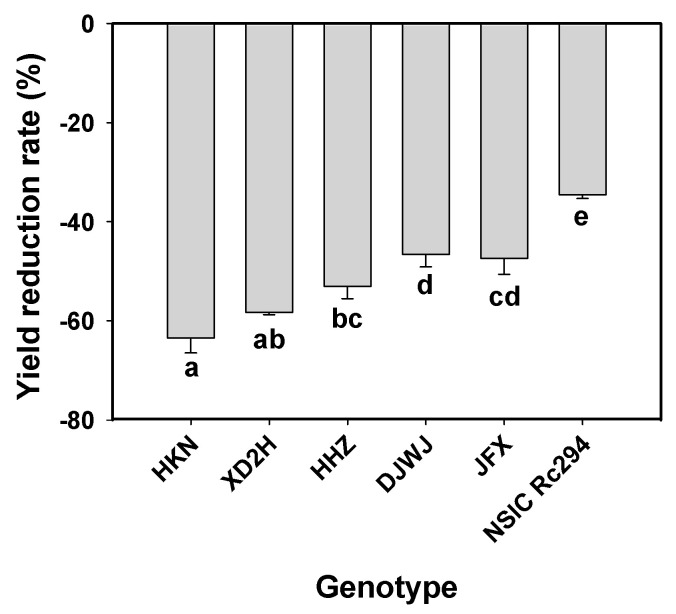
Effect of salt stress on yield reduction rate of different rice genotypes. Different lowercase letters indicate significant difference between genotypes at *p* < 0.05.

Grain yield decreased significantly under salt stress, with more reduction in salt-sensitive rice genotypes (*p* < 0.05, Tables S5 and S6). On average, salt stress reduced the grain yield of salt-tolerant rice by 42.9% and salt-sensitive rice by 58.3% (Table 3, Figure S9). In salt-tolerant rice genotypes, the least yield reduction was shown by NSIC at 34.5%, followed by 47.3% and 46.5% for JFX and DJWJ, respectively. However, in salt-sensitive rice genotypes, the highest yield reduction was produced by HKN at 63.4%, followed by XD2H at 58.2% and HHZ at 53.0% (Figure 10). Overall, salt-sensitive rice genotypes showed 15.3% more yield decline than salt-tolerant rice genotypes under salt treatment (Figure 10).

Under salt stress, both salt-sensitive and salt-tolerant rice genotypes showed an equivalent decrease in panicle number, at 22.0% and 22.8% on average, respectively (Tables S5 and S6). In salt-sensitive rice, XD2H showed the least reduction in panicles at 15.8%, followed by HHZ at 22.8% and HKN at 27.4%. In salt-tolerant rice, NSIC had the minimum decrease in panicles at 18.2%, followed by DJWJ and JFX at 20.6% and 29.5%, respectively (Table 3).

Spikelets per panicle was significantly decreased under salt stress (Tables S5 and S6). On average, the spikelets per panicle of salt-sensitive rice genotypes decreased by 29.9%, and in salt-tolerant rice genotypes, it decreased by 24.8% under salt stress (Table 3). Among these, the salt-sensitive rice of HKN and XD2H and salt-tolerant rice of NSIC showed the highest decreases in spikelets per panicle, at 33.8%, 33.9% and 30.6%, respectively. In addition, the spikelets per panicle of HHZ, DJWJ and JFX showed similar decreases under salt stress, at 22.1%, 23.1% and 20.7%, respectively. It is worth noting that HKN produced the highest spikelets per panicle under both CK and salt stress (Table 3).

The total spikelets was significantly decreased under salt stress (Tables S5 and S6). Overall, there seems to be a small difference in the decline in total spikelets between salt-sensitive and salt-tolerant rice genotypes (3.3% lower in sensitive genotypes) under salt stress (Table 3). On average, the total spikelets of salt-tolerant rice genotypes decreased by 42.1% under salt stress; the lowest decrease of 38.8% was shown by DJWJ, followed by 44.2% and 43.2% for JFX and NSIC, respectively. Similarly, the total spikelets of salt-sensitive rice genotypes decreased by 45.4%; HKN showed the highest decrease at 51.8% under salt stress, followed by XD2H at 44.4% and HHZ at 39.9%. In addition, both HKN and NSIC showed the highest total spikelets under CK and salt stress (Table 3).

The grain-filling percentage was significantly decreased under salt stress (Tables S5 and S6). More reduction was observed in salt-sensitive rice genotypes, which had a 7.4% greater decline in grain-filling percentage compared to salt-tolerant rice genotypes under salt stress (Table 3). On average, the grain-filling percentage of salt-tolerant rice genotypes decreased by 8.5% under salt stress; JFX had the lowest decrease with 6.4%, followed by DJWJ and NSIC with 9.9% and 9.0%, respectively. However, the grain-filling percentage of salt-sensitive rice genotypes decreased by 15.9% under salt stress; XD2H showed the highest decrease of 17.5%, followed by HKN with 15.8% and HHZ with 14.4%. Overall, the salt-tolerant rice genotype NSIC showed the highest grain-filling percentage under both CK and salt treatment (Table 3).

Salt stress had a significant effect on grain weight (*p* < 0.05, Tables S5 and S6). The grain weight of salt-tolerant genotypes was relatively stable under salt stress, with a reduction of 2.6% on average; JFX decreased by only 0.4%, followed by DJWJ and NSIC at 3.5% and 3.8%, respectively. In salt-sensitive rice genotypes, the grain weight decreased by 8.7%; XD2H showed the highest decrease of 9.9%, followed by HKN with 9.1% and HHZ with 7.1%. Overall, salt-sensitive rice genotypes showed a 6.1% higher decrease in grain weight than salt-tolerant rice genotypes under salt treatment (Table 3).

### 2.4. Correlation Analysis

Grain yield, panicle number, total spikelets and grain-filling percentage were correlated positively with fresh weight and dry weight (*p* < 0.05, 0.01 or 0.001, Table 4, Table S7). Contrastingly, grain yield, total spikelets, grain-filling percentage and grain weight were negatively correlated with CAT, MDA and soluble protein (*p* < 0.05, 0.01 or 0.001, Table 4, Table S7). In addition, grain yield was negatively correlated with SOD and POD (*p* < 0.01 or 0.001) but had no correlation with plant height (*p* > 0.05). There was a strong positive correlation between panicle number and plant height (*p* < 0.001), but panicle number was negatively correlated with POD, CAT and MDA (*p* < 0.05 or 0.01). It is interesting to note that tiller number was strongly positively correlated with grain yield and panicles (*p* < 0.01). However, both spikelets per panicle and total spikelets were negatively correlated with SOD and POD (*p* < 0.05, 0.01 or 0.001). Furthermore, there was a negative correlation between spikelets per panicle and soluble protein (*p* < 0.01, Table 4, Table S7). It was shown that Pro content correlated positively with grain yield, spikelets per panicle and total spikelets (*p* < 0.05) but not with panicles, grain filling and grain weight (*p* > 0.05). K^+^ content was positively correlated with grain yield, panicles, spikelets per panicle, total spikelets, grain filling and grain weight (*p* < 0.05, 0.01 or 0.001). On the contrary, K+ content was negatively correlated with grain yield and yield components (*p* > 0.05, Table 4, Table S7).

## 3. Discussion

As one of the most prevalent abiotic stresses, salinity limits crop growth and productivity. In the present study, growth characteristics (plant height, tiller number, dry weight and relative growth rate) of both salt-sensitive and salt-tolerant rice genotypes were significantly decreased under salt stress (Figure 1, Figure 2 and Figure 3, Table 1). Plants’ response to salinity stress is a complex network; in saline soil, rice plants mainly experience osmotic stress, and more Na^+^ accumulates into the plant tissue and eventually rises to toxic levels, which may cause Na^+^ toxicity, thereby reducing water and nutrient acquisition [21,28]. As shown in the present study, Na^+^ content in rice genotypes increased by 456.2% under salt stress. More Na^+^ accumulated in rice plants that were restricted to absorbing K^+^, which led to K^+^ content decreasing by 31.4%. Then, plant growth and development were largely restricted, caused by nutritional imbalances and photosynthesis decline, which led to a prominent decrease in plant height, tiller number, biomass accumulation and relative growth rate, as shown in this study. These results are supported by previous studies [7,15,19,29]. In the literature, it has been proven that the growth traits of rice at tillering significantly affected yield formation during the reproductive stage [30]. As shown in the present study, grain yield, panicle number, total spikelets and grain-filling percentage were correlated positively with fresh weight and dry weight; also, panicle number was strongly positively correlated with plant height (Table 4). In fact, rice genotypes with higher relative growth rates in the vegetative period benefit from accumulating more assimilates in the leaf sheath and culm, which can contribute to achieving a better grain yield [31]. Therefore, a higher crop growth rate with more biomass production can be targeted for improvement when breeding the salt-tolerant rice genotype.

Salinity severely limits grain yield and yield formation of rice. Salt stress can influence all yield components. In the present study, grain yield, panicles, spikelets per panicle, grain-filling percentage and grain weight were all decreased under salt stress. The decline in grain yield and yield components was closely related to the variation in growth traits during the early growth period under salt stress. The number of tillers per hill is one of the most important yield-contributing characteristics; tiller number determines panicle number and affects grain yield. Under salt stress, the tiller number significantly decreased during the tillering stage in rice plants, which produced fewer panicles and caused a yield decrease, as shown in this study. This was supported by the correlation analysis; the tiller number was strongly positively correlated with grain yield and panicles (*p* < 0.01, Table 3). Biomass production has been the main factor contributing to yield increases. In this study, biomass (fresh weight, dry weight) of both salt-sensitive and salt-tolerant rice genotypes was significantly decreased under salt stress, and biomass was positively correlated with grain yield, panicles, total spikelets and grain-filling percentage (*p* < 0.05 or 0.01, Table 3). Biomass production depends on the radiation use efficiency of the crop [32]. The reduction in biomass observed in the present investigation subjected to salinity stress is often associated with a decrease in the rate of photosynthetic capacity due to a lower level of chlorophyll content. This was confirmed by the study of Islam [12] and Abeer [33]; in rice, in their study, salt stress caused a significant decrease in chlorophyll content, which generated a lower biomass production and grain yield. It was suggested that a decrease in chlorophyll content is a first indication of responses in different plants subjected to salinity stress [23]. The serious biomass reduction under salinity leads to a less assimilated transfer to sink (grain), which results in poor grain filling and low grain weight in rice.

Under salinity conditions, the above-mentioned growth traits, yield and yield components showed a greater reduction in salt-sensitive rice genotypes than in salt-tolerant genotypes. The adverse effects of salt stress take place at all levels of rice life, ranging from morphological to molecular levels. Under salt stress, numerous Na^+^ ions accumulate in rice plants to toxic levels, which produces large amounts of reactive oxygen species (ROS) that directly damage nucleic acids and proteins, leading to membrane lipid peroxidation, reduced chlorophyll synthesis and altered enzyme activities in leaves, which eventually causes plant growth reduction and yield loss [3,20]. It was reported that salt-sensitive rice genotypes maintained a high Na^+^/K^+^ ratio, primarily due to higher Na^+^ accumulation than tolerant genotypes under salinity conditions [34]. However, salt-tolerant rice genotypes have stronger Na^+^ exclusion capacity than sensitive genotypes, which can make Na^+^ efflux from the roots to the rhizosphere through the well-recognized SOS1-dependent exclusion system [35]. In addition, lower Na^+^ accumulation in salt-tolerant genotypes results in a low Na^+^/K^+^ ratio, which can induce Na^+^/K^+^ antiporters to regulate Na^+^ sequestration in vacuoles and exclude salts from entering active leaves [11,36]. Then, less damage is exerted on salt-tolerant rice genotypes. This might be one of the reasons for more reduction in growth traits and grain yield in salt-sensitive rice genotypes than in salt-tolerant genotypes under salt stress.

Furthermore, the damage by ROS that are produced under salinity can be mitigated or eliminated by two pathways. One is by the antioxidant enzyme system, increasing the activity of SOD, POD, CAT, etc. Another is the non-enzymatic system, producing osmoregulatory substances of Pro, soluble protein, MDA, etc. [37]. As shown in the present study, SOD, POD, CAT, Pro, soluble protein and MDA were all increased on a large scale under salt stress. The SOD can dismutate O_2_^-^ into H_2_O_2_ and O_2_, and CAT and POD can break down H_2_O_2_ into non-toxic H_2_O and O_2_, thus protecting the plants from damage [38]. Osmotic substances, such as Pro and soluble protein, can neutralize or alleviate the damage of harmful substances [3]. Compared with salt-sensitive rice genotypes, the salt-tolerant genotypes produced 10% higher concentrations of antioxidant enzymes and 25% more soluble protein in the present study. The higher antioxidant enzymes and osmotic substances in salt-tolerant genotypes can effectively protect the membrane system and maintain photosynthetic properties [16]. This indicates that salt-tolerant genotypes have a stronger ability to scavenge ROS or alleviate the damage than salt-sensitive genotypes under salt stress. This was supported by the results of Zhao, etc. [16,39]. This might be another reason why salt-tolerant genotypes do not suffer severe damage from ROS compared to salt-sensitive genotypes under salt stress.

## 4. Materials and Methods

### 4.1. Experimental Design

The experiments were conducted in a greenhouse at the Wenhui Road Campus of Yangzhou University. For the experiments, plastic pots of uniform size (50 cm height × 40 cm diameter) were utilized, with each pot containing 10 kg of sieved paddy surface soil. The soil type in the pots was sandy loam with 21.75 g kg^−1^ organic matter, 1.96 g kg^−1^ total nitrogen, 20.45 mg kg^−1^ available phosphorus, 123 mg kg^−1^ available potassium and pH 6.8.

Six rice genotypes with contrasting responses to salinity were used. These genotypes were screened from our previous study. DJWJ, JFX and NSIC are salt-tolerant, and HKN, XD2H and HHZ are salt-sensitive (Table 5). All the seeds were sown on May 9 and transplanted on 8 June of 2021 and 2022. Two seedlings were sown per hill, and the hill space was 10 × 10 cm. Before transplanting, 2.4 g of pure N (urea) and 0.5 g of P and K compound fertilizer (potassium dihydrogen phosphate (KH_2_PO_4_)) were applied to each pot as the base fertilizer, and 1.2 g and 2.4 g of pure N (urea) were applied 7 days after transplanting and at the panicle initiation stage, respectively. Two salt treatments were arranged as follows: T1 (CK) irrigated with 0 mmol L^−1^ NaCl solution as the control, and T2 with 100 mmol L^−1^ NaCl solution as salt stress. The salt stress was imposed at the seedling stage (14 days after transplanting) for a duration of 7 days. During the treatment period, T1 and T2 were irrigated with 0 mmol L^−1^ NaCl solution and 100 mmol L^−1^ NaCl solution every day to replenish the evaporated water and maintain the water level at 3–5 cm in each pot, respectively. After treatment, all the plants were irrigated with normal irrigation water and maintained with a water level of 3–5 cm until maturity. The study was arranged in a randomized block design with three replicates, and each replicate had 10 pots. Weeds and pests were controlled to prevent yield loss.

### 4.2. Growth Parameter Measurement

Sampling was performed at the seedling stage (early tillering stage) after salt stress, and 10 hills of representative rice plants were selected randomly for each replication. After measuring plant height, tillers and fresh weight, the stems and leaves were separated to determine leaf area. Then, the leaves and stems were dried for 30 min at 105 °C and then dried in an oven at 80 °C to a constant weight to measure the dry weight. The dry weight of the plants was used to calculate the relative growth rate. Relative growth rate (RGR, g plant^−1^ d^−1^) was calculated using the following formula: (W2 − W1)/(T2 − T1), where W1 and W2 represent the measured biomass at T1 and T2 periods, respectively [40].

### 4.3. Physiological Parameter Measurements

After salt stress treatment at the tillering stage, 5 hills of representative plants were selected from each pot, and then the uppermost fully expanded leaves of the main stems were sampled and immersed in liquid nitrogen immediately and then stored in an ultra-low temperature refrigerator (−80 °C) for physiological assay. In this study, the physiological parameters were measured by an automatic microplate reader (Synergy H1, BioTek Instruments, Winooski, VT, USA) to record the absorbance values (OD).

Superoxide dismutase (SOD) activity: 1.0 g of frozen leaves was ground with 3 mL of 0.05 mol L^−1^ PBS buffer (pH = 7.8) and a small amount of quartz sand in an ice bath, then transferred into a 5 mL centrifuge tube and centrifuged at 10,000 r min^−1^ for 15 min at 4 °C. The supernatant was extracted as the enzyme solution. The SOD reaction solution (5 mL 100 mmol L^−1^ potassium phosphate buffer (pH 7.8) containing 0.1 mmol L^−1^ EDTA (ethylenediamine tetraacetic acid disodium salt), 0.1% Triton X-100 and 2% polyvinyl pyrrolidone) and 1 mL enzyme solution were placed in 10 mL centrifuge tubes and immediately subjected to fluorescent tube light at 4000 LX. The reaction was terminated by stopping the light and shading the light after 15 min. The absorbance was measured at 560 nm wavelength (colorimetric assay). One unit of SOD activity is expressed as the amount of enzyme required to cause 50% inhibition of epinephrine oxidation [41].

Peroxidase (POD) activity: About 0.1 g of frozen leaves was ground in 3 mL of 0.1 mol L^−1^ phosphate buffer (pH 7.0) to extract POD. After that, the extraction was centrifuged at 18,000× *g* at 4 °C for 15 min. The supernatant was used as the enzyme source. The oxidized o-diphenylamine was determined at 430 nm. Phosphate buffer (0.1 mol L^−1^, pH 6.5) was placed in colorimetric dishes containing enzyme extract. Then, 0.2 mL 0.2 mol L^−1^ H_2_O_2_ was added and mixed, and the absorbance per min was recorded. The POD activity unit is expressed as the rate of increase in absorbance per min [42].

Catalase (CAT) activity: About 0.1 g of frozen leaves was homogenized in 5 mL assay mixtures, which contained 2.9 mL substrate solution (30% hydrogen peroxide in 50 mmol L^−1^ potassium phosphate buffer) and 0.1 mL of enzyme extract. The decomposition of H_2_O_2_ was stopped by adding 2 mL potassium dichromate (5%) to the mixed solution. The absorbency was measured immediately at 240 nm and read every 30 s for 2 min to calculate the CAT enzyme activity [43].

Malondialdehyde (MDA) content: About 0.5 g of frozen leaves was ground in 0.1% trichloroacetic acid (TCA), then mixed and centrifuged at 12,000× *g* for 15 min to prepare for the MDA extraction. After that, 1 mL supernatant with 4 mL 0.5% thiobarbituric acid (containing 20% trichloroacetic acid) was heated at 95 °C for 15 min and then centrifuged at 10,000× *g* for 15 min. Then, the absorbance of the sample was recorded at 600, 532 and 450 nm, and the MDA content was calculated [44].

Soluble protein content: 0.1 g of frozen leaves was ground and extracted with 5 mL of ice-cold potassium phosphate buffer with 1 mM ascorbic acid (pH 7.8), then centrifuged at 10,000× *g* min^−1^ at 4 °C for 20 min. Then, 0.02 mL of the supernatant was mixed with 5 mL of Kaumas Brilliant Blue G-250 reagent and allowed to sit for 2 min. After that, the absorbance was recorded at 595 nm. The content of soluble protein was calculated using the standard curve [1].

Proline content: 0.5 g of rice leaves was homogenized in 3% sulphosalicylic acid and centrifuged at 10,000× *g* for 15 min. The supernatant, glacial acetic acid, and 140 mM ninhydrin reagent (ratio 1:1:1) were mixed. The mixture was incubated in a water bath at 95 °C for 45 min, and 1 mL toluene was added to the mixture after cooling. The proline content was measured by absorbance at 520 nm. A standard curve was generated for L-proline. The proline content in rice roots was determined from the absorbance and the standard curve [45].

Na^+^ and K^+^: 0.5 g of dry leaves was crushed and digested with HNO_3_ and HClO_4_ (4:1, *v*/*v*) and then concentrated in a microwave oven (Mars, CEM Inc., New York, NY, USA). The final K+ and Na+ concentrations were determined using atomic absorption spectrometry (PinAAcle 900, PerkinElmer Life and Analytical Sciences, Inc., Shelton, DC, USA) [46].

### 4.4. Yield and Yield Component Measurements

At the maturity stage, 50 representative hills of plants in each replicate of treatment were harvested to determine grain yield at 14% moisture. Another 10 hills of plants were sampled from each replicate to determine yield components. After measurement of the plant height and panicles, the grains were threshed manually to calculate the number of spikelets per panicle, filled-grain percentage and 1000-grain weight [5].

### 4.5. Statistical Analysis

The data were collated using Microsoft Excel 2021, and the mean values of each trait were calculated. Statistix 9 software was used for ANOVA and multiple comparisons of the data, and SigmaPlot 10.0 software was used for graphing. Growth parameters were measured using 10 repetitions, and physiological parameters were measured using 5 repetitions. All the parameters were shown as the average values of the 2-year experiments because the tendency of each parameter was similar in each year, and there was no significant difference between the two years. The 2-year experiments data can be downloaded in Supplementary Materials.

## 5. Conclusions

Salt stress inflicted at the tillering stage significantly affects growth, physiological traits and yield formation of both salt-sensitive and salt-tolerant rice. In general, plant height, tiller number, dry weight and relative growth rate showed a greater reduction in salt-sensitive rice genotypes. On the contrary, antioxidant enzyme activity (SOD, POD, CAT) and osmotic adjustment substances (Pro, soluble protein, MDA) significantly increased under salt stress, and the increase was far higher in salt-tolerant rice except for MDA. Consequently, grain yield and yield components significantly decreased under salt stress. Among these, grain yield, spikelets per panicle, grain-filling percentage and grain weight all showed a more severe reduction in salt-sensitive genotypes under salt stress. However, the decrease in panicle number and total spikelets showed a modest gap between salt-sensitive and salt-tolerant rice genotypes. The yield advantage of salt-tolerant rice was partially caused by more biomass accumulation, higher growth rate, and strong antioxidant capacity and osmotic adjustment ability under salt stress.

## Figures and Tables

**Table 1:** Effect of salt stress on fresh weight, dry weight and relative growth rate (RGR) of different rice genotypes.

Variety	Treatment	Fresh Weight (g Plant^−1^)	Dry Weight (g Plant^−1^)	RGR (g Plant^−1^ d^−1^)
HKN	T1	74.8 e	5.8 f	0.11 f
	T2	31.7 h	2.8 g	0.05 g
XD2H	T1	46.5 g	8.5 cd	0.16 c
	T2	36.0 h	3.1 g	0.06 g
HHZ	T1	106.8 d	10.1 b	0.19 b
	T2	59.7 f	6.3 f	0.12 f
DJWJ	T1	172.7 a	10.0 b	0.19 b
	T2	96.1 d	9.0 c	0.17 c
JFX	T1	123.6 c	10.7 ab	0.20 b
	T2	65.3 ef	7.0 e	0.13 e
NSIC Rc294	T1	156.9 b	11.8 a	0.22 a
	T2	67.7 ef	7.9 d	0.15 d
LSD (0.05)		52.678	3.350	0.035
T		***	***	***
V		***	***	***
T × V		**	ns	ns

T1: control, T2: salt treatment; Different lowercase letters indicate significant level of difference between genotypes under different treatments at *p* < 0.05. ns, not significant; ** and *** in the table indicate *p* < 0.01 and 0.001, respectively. The fresh weight, dry weight and RGR were measured with 10 plants in each replicate.

**Table 2:** Effect of salt stress on content of Na^+^, K^+^ and Na^+^/K^+^ in different rice genotypes.

Variety	Treatment	Na^+^ (mg g^−1^ DW)	K^+^ (mg g^−1^ DW)	Na^+^/K^+^ (mg g^−1^ DW)
HKN	T1	1.06 cd	20.0 c	0.053 f
	T2	5.93 a	14.1 f	0.421 c
XD2 H	T1	0.98 d	19.2 cd	0.051 f
	T2	5.57 a	11.4 g	0.489 b
HHZ	T1	1.09 cd	20.6 bc	0.053 f
	T2	5.43 ab	10.8 g	0.503 a
DJWJ	T1	0.85 d	21.1 abc	0.040 g
	T2	4.81 bc	17.5 de	0.275 d
JFX	T1	0.90 d	23.2 a	0.039 g
	T2	4.20 c	16.0 ef	0.263 e
NSIC	T1	0.90 d	22.3 ab	0.040 g
	T2	4.49 c	17.3 de	0.260 h
LSD (0.05)		0.64	2.19	4.98
T		***	***	***
V		**	***	*
T × V		*	*	ns

T1: control, T2: salt treatment; Different lowercase letters indicate significant level of difference between genotypes under different treatments at *p* < 0.05. ns, not significant; *, ** and *** in the table indicate *p* < 0.05, 0.01 and 0.001, respectively.

**Table 3:** Effect of salt stress on grain yield and yield components of different rice genotypes.

Variety	Treatment	Yield (g pot^−1^)	Panicles (pot^−1^)	Spikelets Per Panicle	Total Spikelets (10^3^ pot^−1^)	Grain Filling (%)	1000-Grain Weight (g)
HKN	T1	145.6 a	54.1 c	178.7 a	9.65 a	67.82 d	22.2 a
	T2	52.9 g	39.3 e	118.3 c	4.65 d	57.04 e	20.2 c
XD2H	T1	77.1 f	52.4 c	92.8 d	4.86 d	76.29 b	20.9 c
	T2	32.2 h	44.1 d	61.3 f	2.70 f	62.95 d	19.0 d
HHZ	T1	81.9 e	77.5 a	79.6 e	6.17 c	72.24 c	18.4 e
	T2	38.4 h	59.8 c	62.0 f	3.71 e	61.86 d	17.1 f
DJWJ	T1	102.6 d	77.5 a	89.8 d	6.96 c	73.09 bc	20.2 c
	T2	54.8 g	61.5 b	69.2 f	4.26 d	66.50 d	19.5 d
JFX	T1	111.8 c	70.2 ab	94.1 d	6.61 c	75.68 b	22.5 a
	T2	58.7 g	49.5 d	74.6 e	3.69 e	70.84 c	22.4 a
NSIC	T1	134.4 b	65.3 b	134.9 b	8.81 b	84.34 a	21.0 b
	T2	87.9 e	53.4 c	93.6 d	5.00 d	75.92 b	20.2 c
LSD (0.05)		19.5	6.2	15.1	950.1	0.06	1.2
T		***	***	***	***	***	**
V		***	***	***	***	***	***
T × V		*	ns	**	***	*	ns

T1: control, T2: salt treatment; Different lowercase letters indicate significant difference between genotypes at *p* < 0.05. ns, not significant; *, ** and *** in the table indicate *p* < 0.05, *p* < 0.01 and *p* < 0.001, respectively. The 50 plants were harvested to measure grain yield and an additional 12 plants were harvested to measure yield components in each replicate.

**Table 4:** Correlations of yield and yield components with growth and physiological parameters of different rice genotypes under salt stress.

	Yield	Panicles	Spikelets Per Panicle	Total Spikelets	Grain Filling	1000-Grain Weight
PH	0.3174 ^ns^	0.6338 ***	−0.1033 ^ns^	0.2513 ^ns^	0.3287 ^ns^	0.2793 ^ns^
FW	0.6006 **	0.8373 ***	0.1483 ^ns^	0.6154 **	0.5610 **	0.1410 ^ns^
DW	0.5327 **	0.7736 ***	0.0216 ^ns^	0.4727 *	0.7743 ***	0.1623 ^ns^
TN	0.5801 **	0.6478 ***	0.0791 ^ns^	0.2488 ^ns^	0.2471 ^ns^	−0.0412 ^ns^
SOD	−0.5447 **	−0.2332 ^ns^	−0.4926 *	−0.5818 **	−0.2172 ^ns^	−0.2965 ^ns^
POD	−0.6274 ***	−0.6115 **	−0.4426 *	−0.7003 ***	−0.2726 ^ns^	−0.1744 ^ns^
CAT	−0.5888 **	−0.4629 *	−0.2969 ^ns^	−0.5259 **	−0.4962 *	−0.6022 **
MDA	−0.7049 ***	−0.4687 *	−0.3589 ^ns^	−0.5932 **	−0.6914 ***	−0.5011 *
SPT	−0.7677 ***	−0.2788 ^ns^	−0.5846 **	−0.6963 ***	−0.5606 **	−0.6321 ***
Pro	0.4412 *	0.1342 ^ns^	0.4703 *	0.4843 *	0.1604 ^ns^	0.1432 ^ns^
K^+^	0.8067 ***	0.6207 **	0.451 *	0.749 ***	0.7026 ***	0.5445 **
Na^+^	−0.7974 ***	−0.6494 ***	−0.451 *	−0.7733 ***	−0.6597 ***	−0.4219 *

^ns^, not significant; *, **, *** significant at *p* < 0.05, 0.01 and 0.001. PH: plant height; FW: fresh weight; DW: dry weight; TN: tiller number; SOD: superoxide dismutase; POD: peroxidase; CAT: catalase; MDA: malondialdehyde; SPT: soluble protein; Pro: proline.

**Table 5:** Information on rice genotypes used in this study.

Variety	Abbreviation	Type	Salt Tolerance
Hongkenuo	HKN	japonica	salt-sensitive
Xudao2hao	XD2H	japonica	salt-sensitive
Huanghuazhan	HHZ	indica	salt-sensitive
Dijiaowujian	DJWJ	indica	salt-tolerant
Jiefangxian	JFX	indica	salt-tolerant
NSIC Rc294	NSIC	indica	salt-tolerant

## Data Availability

Data are contained within the article and Supplementary Materials.

## Supplementary Materials

The following supporting information can be downloaded at: https://www.mdpi.com/article/10.3390/plants13081157/s1, Table S1: The ANOVA analysis of plant height and tiller number of rice genotypes under salt stress in 2021 and 2022; Table S2: Effect of salt stress on fresh weight, dry weight and relative growth rate (RGR) of different rice genotypes in 2021 and 2022; Table S3: The ANOVA analysis of MDA, soluble protein (SP) and Proline (Pro) of rice genotypes under salt stress in 2021 and 2022; Table S4: The ANOVA analysis of SOD, CAT and POD of rice genotypes under salt stress in 2021 and 2022; Table S5: Effect of salt stress on grain yield and yield components of different rice genotypes in 2021; Table S6: Effect of salt stress on grain yield and yield components of different rice genotypes in 2022; Table S7: Correlations of yield and yield components with growth and physiological parameters of different rice genotypes under salt stress in 2021; Table S8: Correlations of yield and yield components with growth and physiological parameters of different rice genotypes under salt stress in 2022; Figure S1: Effect of salt stress on plant height of different rice genotypes; Figure S2: Effect of salinity stress on tiller number of different rice genotypes; Figure S3: Effect of salt stress on relative growth rate (RGR) of different rice genotypes; Figure S4: Effect of salt stress on MDA content of different rice genotypes; Figure S5: Effect of salt stress on SOD activity of different rice genotypes; Figure S6: Effect of salt stress on CAT activity of different rice genotypes; Figure S7: Effect of salt stress on POD activity of different rice genotypes; Figure S8: Effect of salt stress on soluble protein of different rice genotypes; Figure S9: Effect of salt stress on yield reduction rate of different rice genotypes.
