# Supplementary material for: Effects of Salt Stress on Physiological and Agronomic Traits of Rice Genotypes with Contrasting Salt Tolerance
Source: Plants (Basel). 2024 Apr 22;13(8):1157. doi: 10.3390/plants13081157 (PMC11054200; doi:10.3390/plants13081157)
Supplement: Supplementary file 1 [file plants-13-01157-s001.zip › plants-2906008-supplementary.pdf]

Table S1. The ANOVA analysis of plant height and tiller number of rice genotypes under salt stress in 2021 and 2022.

Variety	Plant height		Tiller number	
	2021	2022	2021	2022
T	***	***	***	***
V	***	**	**	**
T*V	ns	*	*	*

[ns], not significant; *, **, *** significant at $p < 0.05$, 0.01 and 0.001.

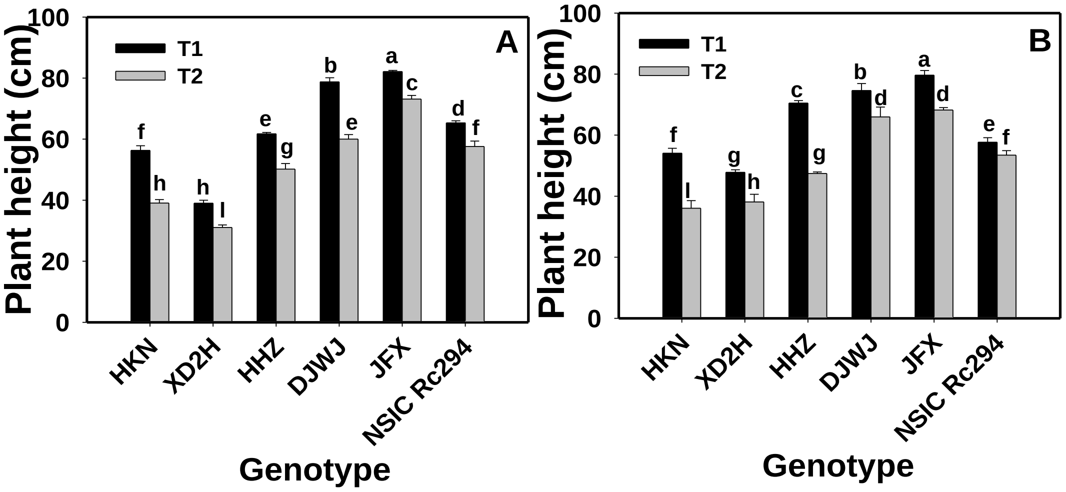

Figure S1. Effect of salt stress on plant height of different rice genotypes. T1: control, T2: salt treatment; Different lowercase letters indicate significant level of difference between genotypes under different treatments at $p < 0.05$. The plant height was measured with 10 repetitions plants in each replicate. A: 2021. B: 2022.

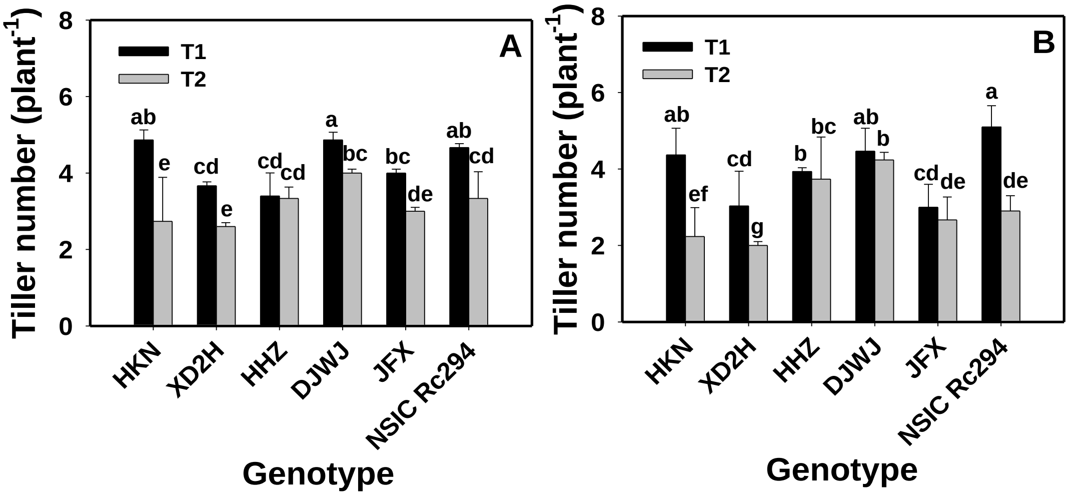

Figure S2. Effect of salinity stress on tiller number of different rice genotypes. T1: control, T2: salt treatment; Different lowercase letters indicate significant level of difference between genotypes under different treatments at $p < 0.05$. The tiller number was measured with 10 repetitions plants in each replicate. A: 2021. B: 2022.

Table S2. Effect of salt stress on fresh weight, dry weight and relative growth rate (RGR) of different rice genotypes in 2021 and 2022.

Variety	Treatment	Fresh weight (g plant^{-1})		Dry weight (g plant^{-1})		RGR (g plant^{-1} d^{-1})	
		2021	2022	2021	2022	2021	2022
HKN	T1	65.8 e	83.9 f	5.1 de	6.5 de	0.08 e	0.14 de
	T2	32.5 h	30.9 k	3.1 e	2.6 g	0.04 f	0.07 f
XD2H	T1	39.2 g	53.9 i	7.8 bc	9.1 bcd	0.13 d	0.19 bc
	T2	25.9 i	46.0 j	3.3 e	2.9 fg	0.04 f	0.07 f
HHZ	T1	96.2 c	117.3 d	9.2 bc	10.9 ab	0.19 ab	0.20 bc
	T2	49.6 f	69.9 g	7.1 cd	5.5 cde	0.11 d	0.12 e
DJWJ	T1	165.9 a	179.5 a	10.2 ab	9.9 abc	0.17 bc	0.21 ab
	T2	97.5 c	94.8 e	8.7 bc	9.4 abc	0.14 cd	0.20 b
JFX	T1	115.1 b	132.2 c	9.4 bc	11.9 a	0.20 ab	0.20 b
	T2	71.2 d	59.4 h	7.7 bcd	6.2 e	0.13 d	0.14 de
NSIC Rc294	T1	166.6 a	147.1 b	12.1 a	11.4 ab	0.21 a	0.24 a
	T2	69.3 de	66.1 g	8.3 bc	7.6 cde	0.14 d	0.16 cd
LSD(0.05)		13.67	4.21	3.42	2.69	0.03	0.03
T		***	***	***	***	***	***
V		***	***	***	***	***	***
T*V		ns	**	ns	ns	***	ns

T1: control, T2: salt treatment; Different lowercase letters indicate significant level of difference between genotypes under different treatments at $p < 0.05$. ^{ns}, not significant; *, ** and *** in the table indicate $p < 0.05$, 0.01 and 0.001, respectively. The fresh weight, dry weight and RGR were measured with 10 repetitions plants in each replicate.

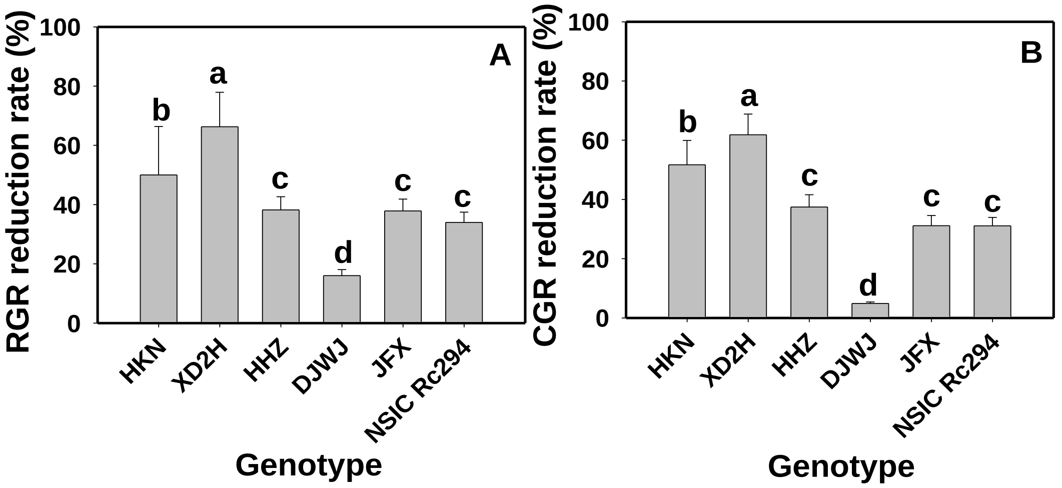

Figure S3. Effect of salt stress on relative growth rate (RGR) of different rice genotypes. Different lowercase letters indicate significant level of difference at $p < 0.05$. The RGR was measured with 10 repetitions plants in each replicate. A: 2021. B: 2022.

Table S3. The ANOVA analysis of MDA, soluble protein (SP) and Proline (Pro) of rice genotypes under salt stress in 2021 and 2022.

Variety	MDA		SP		Pro	
	2021	2022	2021	2022	2021	2022
T	***	***	***	***	***	***
V	***	**	***	***	***	***
T*V	**	*	*	*	**	**

*, **, *** significant at $p < 0.05$, 0.01 and 0.001.

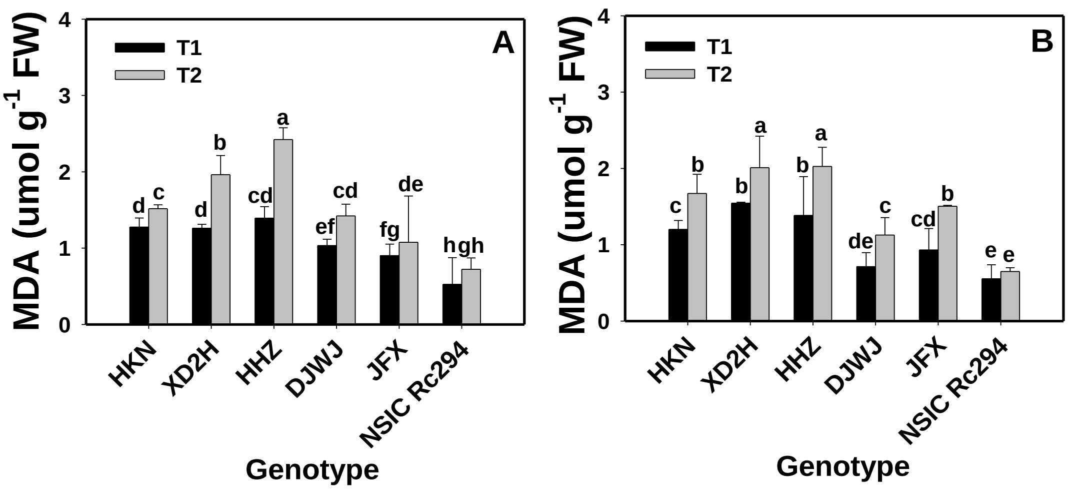

Figure S4. Effect of salt stress on MDA content of different rice genotypes. T1: control, T2: salt treatment; Different lowercase letters indicate significant level of difference between genotypes under different treatment ($p < 0.05$). The MDA was measured with frozen leaves by 5 repetitions in each replicate. A: 2021. B: 2022.

Table S4. The ANOVA analysis of SOD, CAT and POD of rice genotypes under salt stress in 2021 and 2022.

Variety	SOD		CAT		POD	
	2021	2022	2021	2022	2021	2022
T	***	***	***	***	***	***
V	***	**	ns	ns	ns	ns
T*V	ns	*	ns	ns	ns	ns

^{ns}, not significant; *, **, *** significant at $p < 0.05$, 0.01 and 0.001.

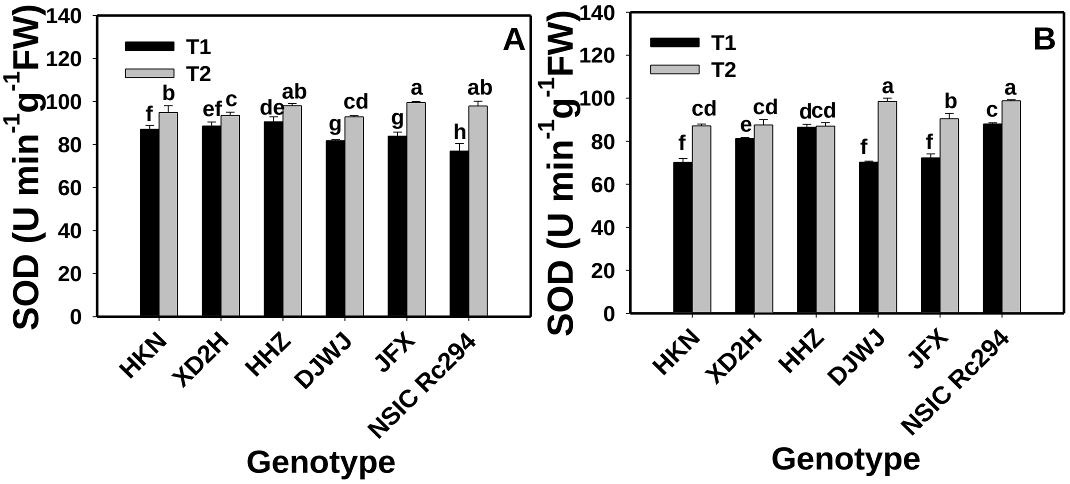

Figure S5. Effect of salt stress on SOD activity of different rice genotypes. T1: control, T2: salt treatment; Different lowercase letters indicate significant differences between genotypes under different treatment (p < 0.05). The SOD was measured with frozen leaves by 5 repetitions in each replicate. A: 2021. B: 2022.

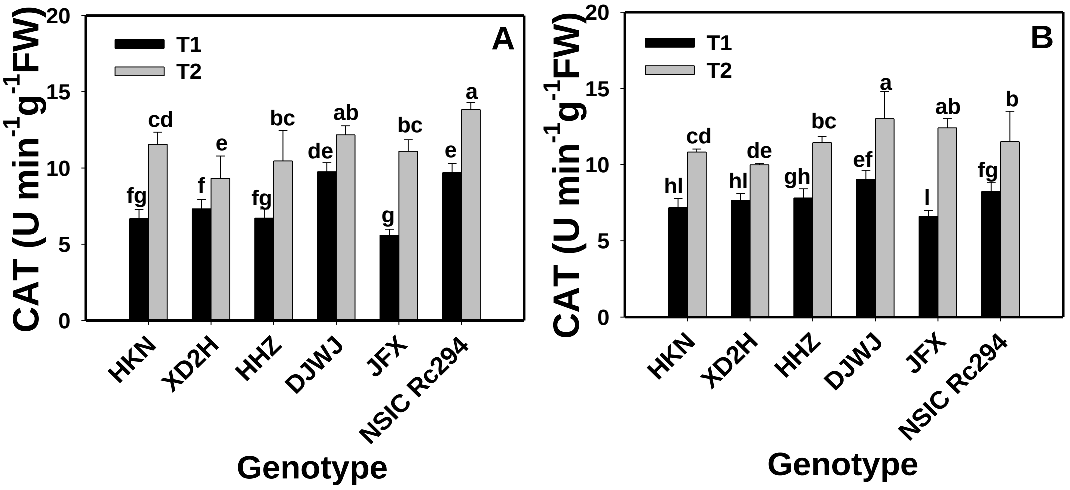

Figure S6. Effect of salt stress on CAT activity of different rice genotypes. T1: control, T2: salt treatment; Different lowercase letters indicate significant level of difference between genotypes under different treatment (p < 0.05). The CAT was measured with frozen leaves by 5 repetitions in each replicate. A: 2021. B: 2022.

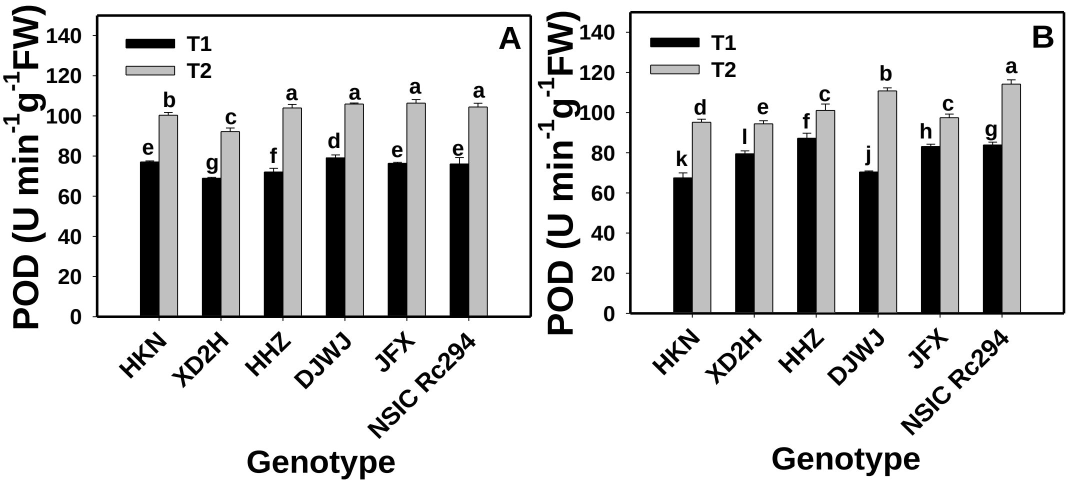

Figure S7. Effect of salt stress on POD activity of different rice genotypes. T1: control, T2: salt treatment; Different lowercase letters indicate significant level of difference between genotypes under different treatment ($p < 0.05$). The POD was measured with frozen leaves by 5 repetitions in each replicate. A: 2021. B: 2022.

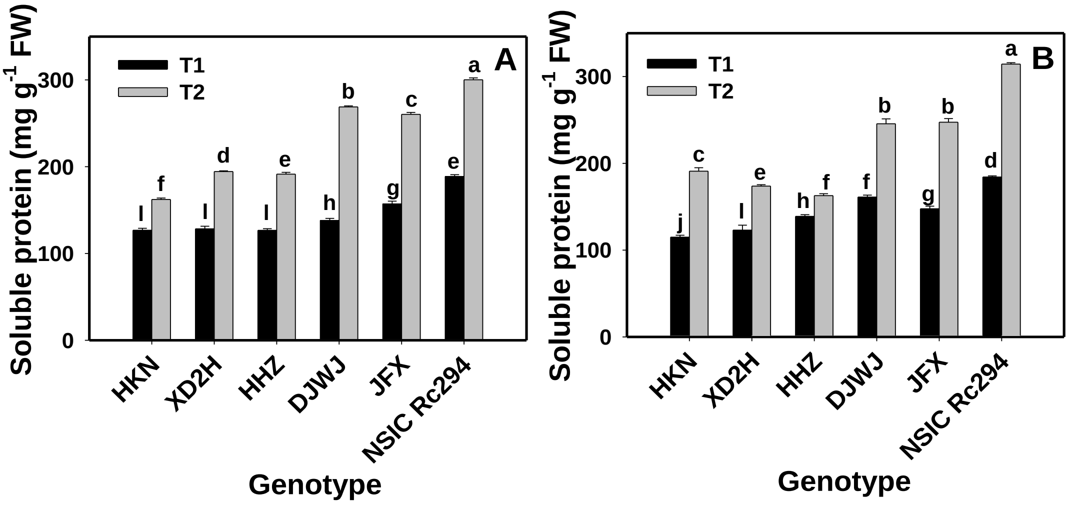

Figure S8. Effect of salt stress on soluble protein of different rice genotypes. T1: control, T2: salt treatment; Different lowercase letters indicate significant level of difference between genotypes under different treatment ($p < 0.05$). The soluble protein was measured with frozen leaves by 5 repetitions in each replicate. A: 2021. B: 2022.

Table S5. Effect of salt stress on grain yield and yield components of different rice genotypes in 2021.

Variety	Treatment	Yield (g pot^{-1})	panicles (pot^{-1})	Spikelets per panicle	Total spikelets (10^3 pot^{-1})	Grain filling (%)	1000-grain weight (g)
HKN	T1	151.5a	51.6e	180.1a	9.31a	67.46bcd	23.1a
	T2	54.8f	39.8g	113.7b	4.42cd	56.04e	19.2cde
XD2H	T1	84.2e	51.7e	97.5bc	4.94cd	74.33ab	20.3bcd
	T2	34.1g	43.1f	56.7f	2.31e	63.62cde	19.5bcd
HHZ	T1	77.8e	76.4a	75.3d	5.67cd	68.87bc	17.6de
	T2	37.3g	53.6e	63.1e	3.24de	59.84de	16.5e
DJWJ	T1	96.1cd	69.9bc	91.9c	6.32bc	67.40bcd	20.0bcd
	T2	52.5f	63.1d	62.5e	3.85de	63.29cde	18.7de
JFX	T1	102.9c	70.7b	87.7cd	6.11bc	67.65bcd	22.1ab
	T2	55.9f	46.2f	71.9d	3.21de	69.04bc	21.7abc
NSIC	T1	141.0b	66.6cd	91.7c	8.68ab	80.12a	19.9bcd
	T2	91.1d	53.7e	96.3bc	5.07cd	73.27ab	20.0bcd
LSD(0.05)		6.81	3.87	33.39	2.79	8.32	2.69
T		***	***	***	***	***	**
V		***	***	***	***	***	***
T*V		***	***	ns	ns	ns	ns

T1: control, T2: salt treatment; Different lowercase letters indicate significant level of difference between genotypes under different treatments at $p < 0.05$. ^{ns}, not significant; *, ** and *** in the table, indicate $p < 0.05$, $p < 0.01$ and $p < 0.001$, respectively. The 50 plants were harvested to measure grain yield and additional 12 plants to measure yield components in each replicate.

Table S6. Effect of salt stress on grain yield and yield components of different rice genotypes in 2022.

Variety	Treatment	Yield (g pot^{-1})	panicles (pot^{-1})	Spikelets per panicle	Total spikelets (10^3 pot^{-1})	Grain filling (%)	1000-grain weight (g)
HKN	T1	139.8a	56.5	177.3a	9.98	68.18de	21.3abc
	T2	51.1g	38.2	122.9c	4.80	58.05f	21.1abc
XD2H	T1	70.1e	52.3	88.1ef	4.73	78.24bc	21.5abc
	T2	30.3i	44.9	65.9h	3.08	62.28ef	18.5de
HHZ	T1	85.6d	78.6	83.9f	6.66	75.61bcd	19.2cde
	T2	39.6h	64.4	60.9h	4.05	63.87ef	17.6e
DJWJ	T1	109.2c	85.1	87.7ef	7.56	78.78bc	20.5bcd
	T2	57.0f	59.9	75.9g	4.63	69.72de	20.2bcd
JFX	T1	120.8b	69.3	100.5d	7.05	83.70ab	22.8ab
	T2	61.4f	52.8	77.3g	4.18	72.63cd	23.2a
NSIC	T1	127.7a	63.4	139.1b	8.84	88.56a	22.1ab
	T2	84.7d	52.2	90.9e	4.87	78.57bc	20.4bcd
LSD(0.05)		5.22	5.63	6.19	2.52	8.43	2.69
T		***	***	***	***	***	**
V		***	***	***	***	***	***
T*V		***	**	***	ns	ns	ns

T1: control, T2: salt treatment; Different lowercase letters indicate significant level of difference between genotypes under different treatments at $p < 0.05$. ^{ns}, not significant; *, **

and *** in the table, indicate $p < 0.05$, $p < 0.01$ and $p < 0.001$, respectively. The 50 plants were harvested to measure grain yield and additional 12 plants to measure yield components in each replicate.

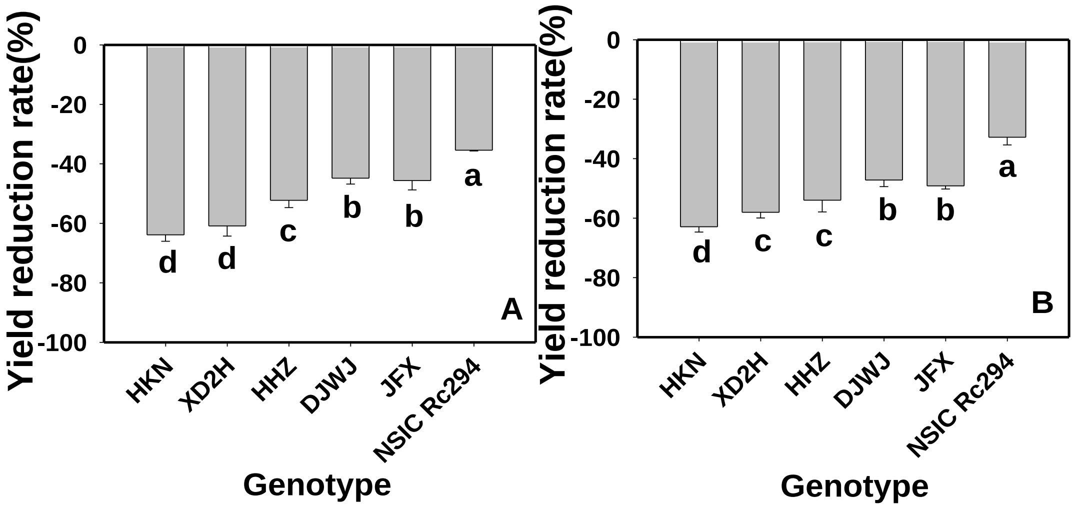

Figure S9. Effect of salt stress on yield reduction rate of different rice genotypes. Different lowercase letters indicate significant difference between genotypes at $p < 0.05$. A: 2021. B: 2022.

Table S7. Correlations of yield and yield components with growth and physiological parameters of different rice genotypes under salt stress in 2021.

	**Yield**	**Panicles**	**Spikelets per panicle**	**Total spikelets**	**Grain Filling**	**1000-grain weight**
PH	0.4197*	0.6906***	0.0046^{ns}	0.3483*	0.2908^{ns}	0.292^{ns}
FW	0.549***	0.789***	-0.0287^{ns}	0.497**	0.4352**	0.1239^{ns}
DW	0.4208*	0.7799***	-0.1972^{ns}	0.5168**	0.7627***	0.2523^{ns}
TN	0.6957***	0.5323***	0.3392*	0.6363***	0.4321**	0.3549*
SOD	-0.7034***	-0.564***	-0.2678^{ns}	-0.5494***	-0.3541*	-0.1536^{ns}
POD	-0.6167***	-0.4886**	-0.324^{ns}	-0.5056**	-0.3851*	-0.2052^{ns}
CAT	-0.4163*	-0.3273^{ns}	-0.3117^{ns}	-0.2883^{ns}	-0.053^{ns}	-0.1848^{ns}
MDA	-0.6438***	-0.338*	-0.3041^{ns}	-0.3517*	-0.3847*	-0.2154^{ns}
SPT	-0.3345*	-0.2256^{ns}	-0.3632*	-0.3529*	0.0505^{ns}	-0.0569^{ns}
Pro	-0.5512*	-0.1232^{ns}	-0.4513*	-0.4943*	-0.1344^{ns}	-0.1634^{ns}
K^+	0.8321***	0.7007**	0.521*	0.7431***	0.7545***	0.5812**
Na^+	-0.7533***	-0.6724***	-0.491*	-0.7813***	-0.6727***	-0.4422*

^{ns}, not significant; *, * *, * ** significant at $p < 0.05$, 0.01 and 0.001. PH: plant height; FW: fresh weight; DW: dry weight; TN: tiller number; SOD: superoxide dismutase; POD: peroxidase; CAT: catalase; MDA: malondialdehyde; SPT: soluble protein.

Table S8. Correlations of yield and yield components with growth and physiological parameters of different rice genotypes under salt stress in 2022.

	**Yield**	**Panicles**	**Spikelets per panicle**	**Total spikelets**	**Grain Filling**	**1000-grain weight**
PH	0.5285***	0.7665***	-0.0613ns	0.3784*	0.6014***	0.3461*
FW	0.6838***	0.8709***	0.1471ns	0.5844***	0.6533***	0.1516ns
DW	0.6175***	0.7022***	0.1038ns	0.5592***	0.9051***	0.4896**
TN	0.5443***	0.5591***	0.3524*	0.6565***	0.5543***	0.2859ns
SOD	-0.5528***	-0.3988**	-0.3936*	-0.4687**	-0.0986ns	-0.1027ns
POD	-0.6451***	-0.3758*	-0.5312***	-0.5867***	-0.2165ns	-0.1737ns
CAT	-0.6063***	-0.2973ns	-0.4459**	-0.5002**	-0.3028ns	-0.0649ns
MDA	-0.7097***	-0.3632*	-0.3917*	-0.4215*	-0.5369***	-0.217ns
SPT	-0.2897ns	-0.296ns	-0.2915ns	-0.3567*	0.0245ns	0.0904ns
Pro	-0.4112*	-0.1514ns	-0.4923*	-0.4763*	-0.1814ns	-0.1314ns
K^+	0.8113***	0.6104**	0.421*	0.7801***	0.6845***	0.5232**
Na^+	-0.8342***	-0.6227***	-0.423*	-0.7524***	-0.6347***	-0.4121*

ns, not significant; *, * *, * ** significant at $p < 0.05$, 0.01 and 0.001. PH: plant height; FW: fresh weight; DW: dry weight; TN: tiller number; SOD: superoxide dismutase; POD: peroxidase; CAT: catalase; MDA: malondialdehyde; SPT: soluble protein; Pro: proline.
